# Hospitals of the World

**Published:** 1893-04-15

**Authors:** 


### HOSPITALS OF THE WORLD.*

(*Continued from page* 416, *Vol. XIII.*)

### IV.—NURSING SYSTEMS ABROAD.

In the past, and to a great extent at the present time, the nursing of the rich on the Continent has been in the hands of religious Sisterhoods, especially the Sisters of St. Vincent de Paul. That order, founded in 1633, is the largest order of nursing nuns. Madame Le Gras was the first Superior and she obtained permission to work in the Hotel Dieu, visiting the patients, reading and praying with them, and providing them with extra nourishment. Some amount of training seems early to have been afforded to the Sisters, since in 1664 we find a woman who purposes becoming a nurse seeking instruction at the Hotel Dieu. "Caterine Barbusseau came to ask the company to permit her to learn bleeding in the Hotel Dieu." In 1789 the Order of St. Vincent had 426 houses in France, and some in Poland, Austria and Silesia. In 1847 the Sisters numbered between 6,000 and 7,000, and were working in Smyrna, Alexandria, and other remote localities. They are admitted to the order without dower after six months' probation and five years' novitiate. Very notable are the words of St. Vincent de Paul in founding this uncloistered order: "Your convent must be the hospital; your cell, the ward; your chapel, the parish church; your cloister, the streets of the city; your rule, the vow of obedience; your grille, the fear of God; your veil, holy modesty." Many other communities of nurses did good service in nursing the sick in France. During the Revolution 255 communities were dissolved, but exceptions were made even during the Reign of Terror in favour of the nursing sisters. The great crisis in the French nursing world occurred after 1877, when the Municipal Council of Paris instituted a Municipal School for Nurses, male and female. This was the preliminary to replacing the Sisters at the Parisian hospitals by lay nurses, trained in theoretical and practical classes. The measure was not a success; it caused much bitterness of spirit and discontent among the patients, and great pain to the Sisters, whose services were discarded. The new nurses were recruited from the ranks of those who had not succeeded in getting employment elsewhere, and behaved as might have been expected. They drank the patients' wine, they demanded tips, and they are even accused of having drunk the spirit in which the doctors preserved their specimens! In 1889 the decree banishing the Sisters was repealed. They now work in conjunction with lay nurses, and under improved methods of training and organisation. The story of two nurses who were presented in 1889 with the ribbon of the Legion of Honour proves that heroism is not confined to one party alone. Madame Coralie Cahen was the widow of a doctor, and joined the Society for the Help of the Wounded when the Franco-German war broke out. She worked with great devotion and fearlessness, nursing members of both armies. But she would permit no insult to France, and when the German officers hoisted their flag over her ambulance at Vendôme, after the capitulation of Metz, she threatened to depart with her staff and leave the 800 sick to their fate. The German officers, admiring her patriotism, replaced the French flag. After the war she travelled through Germany, seeking out her countrymen who were detained in prisons or hospitals, and was presented by the Empress Augusta with the Red Cross of the German Order. The services of Sister Maria Theresa were of an even higher order. She was but 20 when she nursed the wounded at Balaclava, and was wounded herself. She was wounded again at Magenta. She nursed the wounded in wars in Syria, China, and Mexico. She was carried wounded off the field at Wörth, and was performing her duties again before she fully recovered. Finally, when a grenade fell into her ambulance, she took it in her hands and fearlessly carried it to a distance of a hundred yards, where it exploded and inflicted upon her severe injuries. The general in command might well say that no soldier has ever performed his duty more heroically and lived more successfully for his country.

In Germany a very complete nursing system has been developed by means of several distinct nursing societies for women. The Fatherland Ladies' Society numbers over 64,500 members. The Clement House, the Ladies' Lazaret Society, and the Victoria Home, Berlin, all receive probationers and give training to nurses, who are sent afterwards to work in institutions or private families. The Roman Catholic communities have also many orders nursing in Germany, and a slowly increasing number of Brothers of Charity are devoted to the care of the sick. The Deaconess Institute at Kaiserswerth, on the Rhine, where Miss Nightingale and many of the best English and American nurses have been trained, was opened by Pastor Fliedner in 1836. The fame of the Kaiserswerth nursing system has extended all over the world. The hospital is divided into four departments—men, boys, women, and children. There

* "Hospitals and Asylums of the World," 4 vols., and Portfolio of Plans. (Messrs. Churchill and the Scientific Press.)

is no resident medical man. The period of training is three years. There is no payment until after the first six months, and the salary given later is never more than sufficient to provide clothing. In sickness and old age the Mother House cares for her Sisters, so that high wages are not deemed necessary. In spite of the high standard of nursing in most German hospitals, the wards are said to be greatly wanting in that air of refinement and cheerful comfort which so often in English hospitals impresses the visitors.

In Italy, in spite of the suppression of the religious orders, the nursing is still to a great extent in the hands of Sisters. The famous Maggiore Hospital at Milan is worked by the Sisters of St. Vincent de Paul, officially called "sorveglianti' (superintendents). In many Italian hospitals the nurses are entirely untrained, but the present Queen of Italy is interesting herself much in the question of hospitals, and the nursing systems will probably before long receive attention. In Florence for centuries past excellent work has been done by the Brothers of the Misericordia. These Brothers belong to all ranks of society, married and single, and are bound by the single vow to lend their services freely when demanded to the sick and poor of the town. Their names are not known, and the distinctive dress which hides the whole face and person leaving only the eyes free, prevents their being recognised when on duty.

In Spain, the hospitals for women are nursed by nuns, those for men by monks. Sisters also nurse the rich in their own homes, but the poor are terribly neglected. By Royal decree a room is set apart in every hospital for the treatment of non-Catholics, and the very issuing of such a decree marks the unworthy distinction observed, which causes Protestant patients to feel themselves in a marked manner unacceptable to the Sisters.

There are many nursing schools in America, and the training given in the best of them is in many, if not most respects, similar to that afforded in England. It is noted as an innovation that the nurses at St. Luke's, Chicago, have lately adopted an outdoor uniform of grey cloak, bonnet, and veil. There are no nursing institutions in America, the women there are too self-reliant and jealous of personal liberty not to receive their own earnings. The institution is replaced in every town by a nurses' registry or directory. The directory issues an application for nurses, which requires a statement of the name, age, address, qualifications, kind of nursing preferred, rates of charge, and both medical and family references. If all is satisfactory, the nurse pays five dollars and is registered. In addition, after each engagement is ended, detailed inquiries are sent both to the physician and the family, so that a continuance of the nurse's character and skill may be ensured. The doctor, or person requiring a nurse, applies at the directory, pays a fee of one dollar, and is supplied with a suitable nurse's address. The directory does not attempt to control the rates charged by the nurses, except that they are not allowed to charge more than the rate at which they have registered themselves. At first these moderate fees of nurses and patients did not pay expenses, but as the work was expanded it has become a financial success. American nurses appear often to make large incomes, but the life is a lonely one, and lacking in all the charm of associated interest which binds the members of many English nursing homes so happily together.

